# The gut-brain-metabolic axis: exploring the role of microbiota in insulin resistance and cognitive function

**DOI:** 10.3389/fmicb.2024.1463958

**Published:** 2024-11-26

**Authors:** Gulshara Zh Abildinova, Valeriy V. Benberin, Tamara A. Vochshenkova, Alireza Afshar, Nadiar M. Mussin, Asset A. Kaliyev, Zhanna Zhussupova, Amin Tamadon

**Affiliations:** ^1^Gerontology Center, Medical Center Hospital of the President's Affairs Administration of the Republic of Kazakhstan, Astana, Kazakhstan; ^2^Corporate Fund “Institute for Innovational and Profilaxy Medicine”, Astana, Kazakhstan; ^3^Department of Surgery No. 2, West Kazakhstan Medical University, Aktobe, Kazakhstan; ^4^Department of Neurology, Psychiatry and Narcology, West Kazakhstan Marat Ospanov Medical University, Aktobe, Kazakhstan; ^5^Department of Natural Sciences, West Kazakhstan Marat Ospanov Medical University, Aktobe, Kazakhstan; ^6^Stem Cells Technology Research Center, Shiraz University of Medical Sciences, Shiraz, Iran; ^7^PerciaVista R&D Co., Shiraz, Iran

**Keywords:** brain-gut axis, microbiota, insulin resistance, cognition, hypothalamo-hypophyseal system, volatile fatty acids, neuroinflammatory diseases, neuronal plasticity

## Abstract

The gut-brain-metabolic axis has emerged as a critical area of research, highlighting the intricate connections between the gut microbiome, metabolic processes, and cognitive function. This review article delves into the complex interplay between these interconnected systems, exploring their role in the development of insulin resistance and cognitive decline. The article emphasizes the pivotal influence of the gut microbiota on central nervous system (CNS) function, demonstrating how microbial colonization can program the hypothalamic–pituitary–adrenal (HPA) axis for stress response in mice. It further elucidates the mechanisms by which gut microbial carbohydrate metabolism contributes to insulin resistance, a key factor in the pathogenesis of metabolic disorders and cognitive impairment. Notably, the review highlights the therapeutic potential of targeting the gut-brain-metabolic axis through various interventions, such as dietary modifications, probiotics, prebiotics, and fecal microbiota transplantation (FMT). These approaches have shown promising results in improving insulin sensitivity and cognitive function in both animal models and human studies. The article also emphasizes the need for further research to elucidate the specific microbial species and metabolites involved in modulating the gut-brain axis, as well as the long-term effects and safety of these therapeutic interventions. Advances in metagenomics, metabolomics, and bioinformatics are expected to provide deeper insights into the complex interactions within the gut microbiota and their impact on host health. Overall, this comprehensive review underscores the significance of the gut-brain-metabolic axis in the pathogenesis and treatment of metabolic and cognitive disorders, offering a promising avenue for the development of novel therapeutic strategies targeting this intricate system.

## 1 Introduction

The gut-brain-metabolic axis represents a complex and bidirectional communication network that integrates the gut microbiota, the central nervous system (CNS), and metabolic processes (Zhang Q. et al., 2022). Emerging evidence suggests that the gut microbiota plays a crucial role in modulating insulin resistance and cognitive function through various mechanisms (Zhang Q. et al., 2022; Takeuchi et al., 2023). Understanding the interplay between the gut microbiota, insulin resistance, and cognitive function is crucial for developing targeted therapeutic interventions to prevent and treat metabolic and neurodegenerative disorders (Fekete et al., 2024).

The gut microbiota can influence brain function and metabolic processes through neural, endocrine, and immune pathways (Makris et al., 2021). Microbial metabolites, particularly short-chain fatty acids (SCFAs), have been shown to modulate inflammation, enhance gut barrier integrity, and promote neurogenesis (Loh et al., 2024). Butyrate, a SCFA produced by gut bacteria, has been found to improve insulin sensitivity and protect against neuroinflammation (Li et al., 2022).

Insulin resistance in the brain, often referred to as brain insulin resistance, has been associated with cognitive decline and neurodegenerative diseases like Alzheimer’s disease (Ye et al., 2022). Impaired insulin signaling in the brain leads to decreased glucose uptake, increased oxidative stress, and neuroinflammation, contributing to synaptic dysfunction and neuronal loss (Martinez-Montoro et al., 2022). Gut microbiota dysbiosis has been linked to increased intestinal permeability and systemic inflammation, which can exacerbate insulin resistance and cognitive impairment (Rubinstein et al., 2023).

Therapeutic interventions targeting the gut-brain-metabolic axis, such as probiotics, prebiotics, dietary modifications, and fecal microbiota transplantation (FMT), have shown promise in improving insulin sensitivity and cognitive function (Kolobaric et al., 2024). However, further research is needed to optimize these interventions, understand their long-term effects, and identify the most effective strategies for different populations (Zheng et al., 2023).

Despite of all of the knowledge in this field of study, the majority of the existing research has focused on the general population or specific disease states, such as metabolic disorders and neurodegenerative diseases (Matheson and Holsinger, 2023). However, the role of the gut-brain-metabolic axis in other conditions, including psychiatric disorders and neurological diseases, remains largely unexplored. Expanding the scope of research to investigate the broader implications of this axis could lead to the development of more personalized and effective therapeutic strategies (Loh et al., 2024).

Current research on the gut-brain-metabolic axis is diverse, focusing on various mechanisms through which gut microbiota influence insulin resistance and cognitive function (Fekete et al., 2024). For instance, it has demonstrated the role of SCFAs in modulating inflammatory pathways, while other research highlights the influence of gut-derived neurotransmitters on cognitive processes (Silva et al., 2020). However, the landscape of research remains fragmented, with varying methodologies and outcomes across different studies, leading to ongoing debates about the precise mechanisms at play. As such, it is essential to approach conclusions with caution and recognize the need for further investigation to build a comprehensive understanding of these interactions.

This review aims to provide a comprehensive analysis of the role of gut microbiota in insulin resistance and cognitive function, highlighting the underlying mechanisms and potential therapeutic targets. By elucidating the complex interactions within the gut-brain-metabolic axis, we can pave the way for personalized approaches to prevent and treat metabolic and neurodegenerative disorders.

## 2 Gut-brain axis: mechanisms of interaction

### 2.1 Gut microbiota and the central nervous system

The gut-brain axis facilitates bidirectional communication between the gut microbiota and the CNS through neural, endocrine, and immune pathways (Forsythe and Kunze, 2013).

#### 2.1.1 Neural pathways

The vagus nerve plays a pivotal role in the neural pathway, serving as the primary conduit for communication between the gut and the brain (Breit et al., 2018). Through afferent sensory fibers, the vagus nerve detects changes in the gut environment, such as microbial composition and nutrient availability, and transmits these signals to the brainstem (Gershon and Margolis, 2021). Studies have shown that activation of the vagus nerve can modulate mood and behavior, influencing anxiety and depression (Breit et al., 2018). Additionally, gut-derived metabolites, such as SCFAs, can influence vagal afferent signaling, further linking gut microbiota to neural pathways (Silva et al., 2020).

#### 2.1.2 Endocrine pathways

The hypothalamic–pituitary–adrenal (HPA) axis is central to the endocrine communication between the gut and brain (Rusch et al., 2023). Stress-induced activation of the HPA axis leads to the release of cortisol, which can affect gut permeability and alter microbial composition (Rusch et al., 2023). In turn, certain gut microbes can modulate the release of hormones like ghrelin and leptin, which regulate appetite, energy homeostasis, and even cognitive function (Han et al., 2021). For example, microbial metabolites such as SCFAs and bile acids can influence hormone secretion by interacting with enteroendocrine cells in the gut (Masse and Lu, 2023).

#### 2.1.3 Immune pathways

Immune signaling is another crucial component of gut-brain communication (Kasarello et al., 2023). The gut-associated lymphoid tissue (GALT) plays a key role in immune surveillance and responds to microbial antigens (Kasarello et al., 2023). Cytokines released by immune cells in the gut can cross the blood–brain barrier or signal through the vagus nerve, influencing neuroinflammation and CNS function (Kasarello et al., 2023). Dysbiosis, or microbial imbalance, has been linked to an increase in pro-inflammatory cytokines, which can contribute to neuroinflammatory conditions such as Alzheimer’s disease and depression (Leblhuber et al., 2021).

The vagus nerve is a primary conduit for these signals, transmitting information from the gut to the brain and vice versa. Gut microbiota can influence brain function by producing neurotransmitters such as serotonin, dopamine, and gamma-aminobutyric acid (GABA), which can cross the blood–brain barrier and affect mood, cognition, and behavior (Cryan and Dinan, 2012).

Gut microbiota also affects the HPA axis, a major stress response system (Rusch et al., 2023). Dysbiosis can lead to altered HPA axis activity, resulting in changes in cortisol levels that impact both metabolic and cognitive functions (Misiak et al., 2020). Changes in HPA axis activity can be assessed through various methods, such as measuring cortisol levels in saliva, blood, or hair samples (Wright et al., 2015). These assessments provide insight into the relationship between dysbiosis and stress response, highlighting how disruptions in gut microbiota can influence cortisol production, which in turn affects metabolic and cognitive health (Rogers et al., 2016). Long-term alterations in cortisol levels due to dysbiosis may contribute to insulin resistance, inflammation, and impaired cognitive function (Rogers et al., 2016). Additionally, microbial metabolites such as SCFAs play a critical role in modulating inflammation and maintaining the integrity of the blood–brain barrier, which is crucial for preventing neuroinflammation and preserving cognitive function (Braniste et al., 2014).

The bidirectional communication between the gut microbiota and the host involves complex neural, endocrine, and immune pathways, each playing distinct roles in modulating metabolic and cognitive functions:

#### 2.1.4 Neural pathways

The gut-brain axis facilitates communication between the gut microbiota and the central nervous system (CNS) through the vagus nerve (Carabotti et al., 2015). This pathway enables the transmission of signals, such as neurotransmitters (e.g., serotonin and dopamine), produced by gut microbiota, which can influence mood, cognition, and behavior (Chen et al., 2021). Additionally, gut microbiota can affect the release of neurotrophic factors that support neuronal health and synaptic plasticity, essential for learning and memory (Glinert et al., 2022).

#### 2.1.5 Endocrine pathways

Gut microbiota interact with the endocrine system by influencing the secretion of various gut hormones, including glucagon-like peptide-1 (GLP-1) and peptide YY (PYY) (Zeng et al., 2024). These hormones not only regulate appetite and glucose homeostasis but also have neuroprotective effects that can enhance cognitive function (Suzuki et al., 2011). Dysregulation of these hormones due to altered gut microbiota composition has been linked to insulin resistance and cognitive decline (Patra et al., 2023).

#### 2.1.6 Immune pathways

The gut microbiota plays a crucial role in modulating the immune system, particularly through the production of SCFAs that promote anti-inflammatory responses (Wu and Wu, 2012). These metabolites can influence systemic inflammation, which is a contributing factor in both insulin resistance and neuroinflammation (Vinuesa et al., 2021). Furthermore, the immune response triggered by gut microbiota can affect the permeability of the blood–brain barrier, thereby impacting the communication between the gut and the CNS (Tang et al., 2020).

By elucidating these pathways, we can better understand the mechanisms through which gut microbiota influence both metabolic and cognitive health. Future research should focus on the interplay between these pathways to develop targeted interventions that can optimize health outcomes.

### 2.2 Role of microbial metabolites

Microbial metabolites, particularly SCFAs like acetate, propionate, and butyrate, are produced through the fermentation of dietary fibers by gut bacteria (Abdelhalim, 2024). SCFAs have significant effects on host metabolism and brain function (Qian et al., 2022). Butyrate, for instance, has anti-inflammatory properties and can enhance insulin sensitivity by activating peroxisome proliferator-activated receptor gamma (PPAR-*γ*) and inhibiting histone deacetylases (HDACs), which regulate gene expression involved in glucose and lipid metabolism (Matheus et al., 2017).

Butyrate also promotes neurogenesis and protects against neuroinflammation by inhibiting nuclear factor-kappa B (NF-κB) signaling, a key pathway in inflammatory responses (Alpino et al., 2024). This neuroprotective effect is crucial in maintaining cognitive function and preventing neurodegenerative diseases. Additionally, SCFAs can influence gut-brain signaling through the gut hormone system, including the release of peptides like glucagon-like peptide-1 (GLP-1) and peptide YY (PYY), which regulate appetite and glucose homeostasis (Lai et al., 2024).

## 3 Dysbiosis and insulin resistance

### 3.1 Mechanisms of dysbiosis-induced insulin resistance

**Figure 1:**
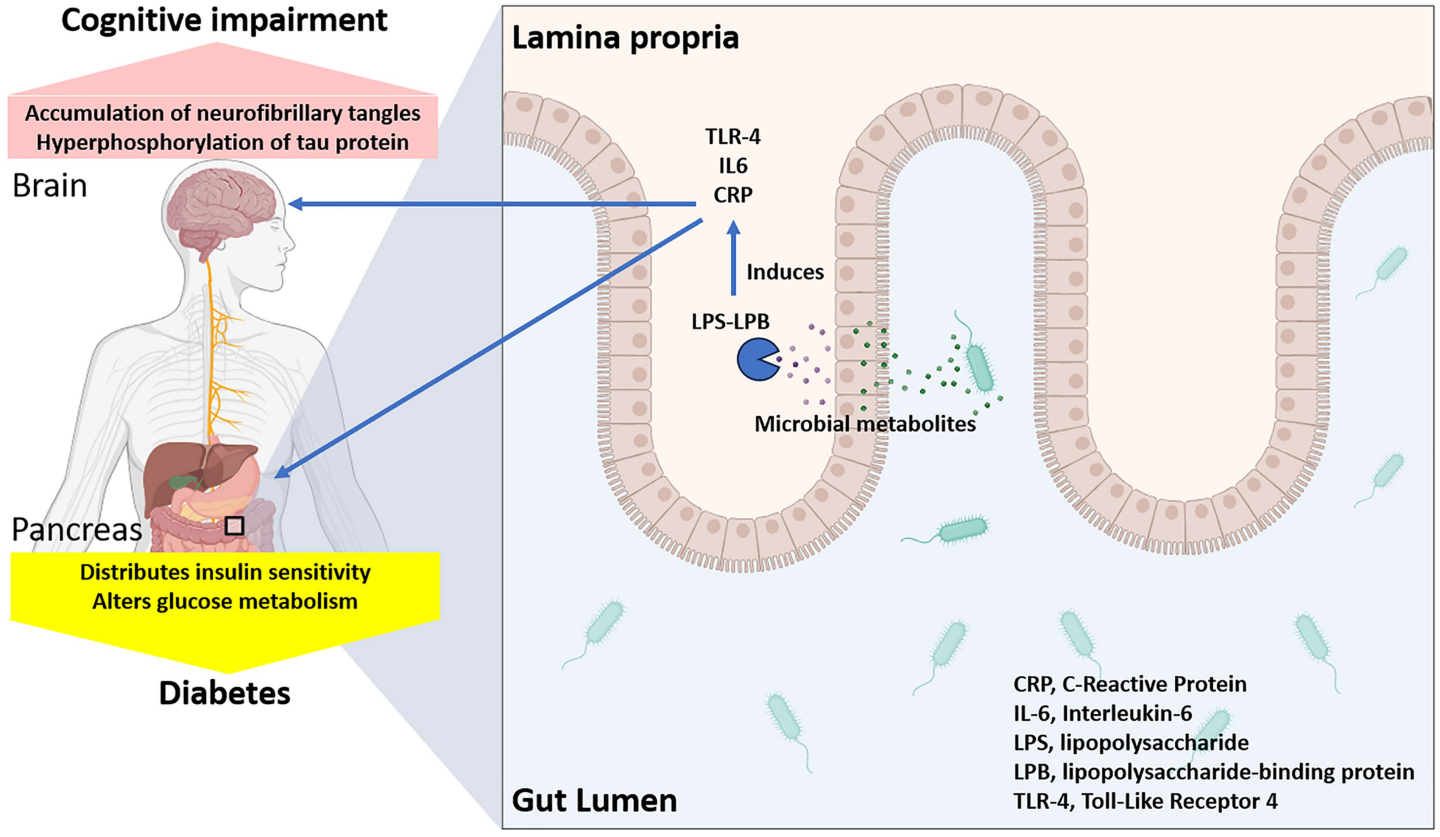
Gut dysbiosis and increased intestinal permeability leading to disrupted glucose metabolism and cognitive decline.

Dysbiosis, characterized by a reduced diversity and altered composition of gut microbiota, has been strongly associated with the development of insulin resistance (Abildinova et al., 2024). For example, a study demonstrated that individuals with type 2 diabetes had significantly lower levels of Firmicutes and increased Bacteroidetes, suggesting a microbial imbalance linked to insulin resistance (Craciun et al., 2022). Another study identified specific gut microbial markers associated with insulin resistance and type 2 diabetes, further supporting the link between dysbiosis and metabolic dysfunction (Wu et al., 2023). One primary mechanism involves increased intestinal permeability, often referred to as “leaky gut” (Vanuytsel et al., 2021). Dysbiosis can disrupt the gut barrier function, allowing the translocation of lipopolysaccharides (LPS) from gram-negative bacteria into the bloodstream, leading to metabolic endotoxemia (Violi et al., 2023). This endotoxemia triggers systemic inflammation, which impairs insulin signaling and promotes insulin resistance (Huang et al., 2021; Figure 1).

Inflammatory cytokines such as tumor necrosis factor-alpha (TNF-*α*) and interleukin-6 (IL-6) play a crucial role in this process by activating serine kinases that phosphorylate insulin receptor substrates, thereby inhibiting their ability to transmit insulin signals (Amabebe et al., 2020). In addition to TNF-α and IL-6, other pro-inflammatory cytokines such as interleukin-1β (IL-1β) also contribute to insulin resistance by exacerbating inflammatory pathways (Al-Mansoori et al., 2022). These cytokines interact in a complex network, amplifying each other’s effects through feedback loops that further promote inflammation (Megha et al., 2021). For instance, TNF-α can induce the production of IL-6 and IL-1β, which in turn perpetuate inflammation by activating downstream signaling pathways such as nuclear factor-kappa B (NF-κB) (Brasier, 2010). This persistent inflammatory state not only disrupts insulin signaling but also contributes to systemic metabolic dysfunction, highlighting the intricate and multifaceted role of cytokines in insulin resistance (Rehman and Akash, 2016). Moreover, dysbiosis-induced alterations in bile acid metabolism can influence insulin sensitivity (Patra et al., 2023). Bile acids act as signaling molecules that modulate metabolic processes through their receptors, such as farnesoid X receptor (FXR) and G protein-coupled bile acid receptor 1 (TGR5) (de Aguiar Vallim et al., 2013). Dysbiosis can disrupt bile acid homeostasis, leading to impaired insulin signaling and glucose metabolism.

### 3.2 Impact of gut microbiota on glucose metabolism

Gut microbiota influence glucose metabolism through various pathways, including the regulation of gluconeogenesis and glycolysis in the liver (Utzschneider et al., 2016). Certain gut bacteria, such as *Akkermansia muciniphila*, have been shown to improve glucose tolerance and insulin sensitivity by modulating mucin production and enhancing gut barrier integrity (Xu et al., 2020). Additionally, gut microbiota can affect the expression of glucose transporters in the intestinal epithelium, influencing glucose absorption and utilization (Ridaura et al., 2013).

Studies have demonstrated that FMT from lean donors to individuals with metabolic syndrome can improve insulin sensitivity, highlighting the potential of gut microbiota manipulation as a therapeutic strategy for insulin resistance (Kootte et al., 2017). Furthermore, dietary interventions that promote beneficial gut bacteria, such as high-fiber diets, have been shown to improve glycemic control and reduce the risk of type 2 diabetes (Deehan and Walter, 2016).

## 4 Insulin resistance and cognitive function

### 4.1 Insulin signaling in the brain

Insulin plays a crucial role in brain function, influencing synaptic plasticity, neurotransmitter release, and neuronal survival (Kleinridders et al., 2014). Insulin signaling in the brain is mediated through the insulin receptor (IR) and insulin-like growth factor-1 receptor (IGF-1R), which activate downstream pathways such as phosphoinositide 3-kinase (PI3K)/Akt and mitogen-activated protein kinase (MAPK) (Ferreira et al., 2018). These pathways are essential for maintaining cognitive functions, including learning and memory.

Impaired insulin signaling in the brain, often referred to as brain insulin resistance, has been associated with cognitive decline and neurodegenerative diseases like Alzheimer’s disease (Escobar et al., 2022). Brain insulin resistance leads to decreased glucose uptake and utilization, increased oxidative stress, and neuroinflammation, all of which contribute to synaptic dysfunction and neuronal loss (Arnold et al., 2018).

### 4.2 Impact of insulin resistance on cognitive function

Insulin resistance is linked to cognitive impairment through several mechanisms (Tyagi and Pugazhenthi, 2021). Hyperinsulinemia, a compensatory response to peripheral insulin resistance, can lead to reduced insulin transport across the blood–brain barrier, resulting in decreased insulin availability in the brain (Vogelsang et al., 2018). This deficiency impairs glucose metabolism and energy production in neurons, contributing to cognitive deficits.

Additionally, insulin resistance is associated with increased amyloid-beta (Aβ) production and decreased clearance, a hallmark of Alzheimer’s disease pathology (Escobar et al., 2022). Insulin can modulate the activity of enzymes involved in Aβ production and degradation, and impaired insulin signaling exacerbates Aβ accumulation and neurotoxicity. Moreover, insulin resistance is linked to tau hyperphosphorylation, another key feature of Alzheimer’s disease, through the dysregulation of glycogen synthase kinase-3β (GSK-3β) (Rodriguez-Rodriguez et al., 2017).

## 5 Microbial metabolites and cognitive function

### 5.1 Role of SCFAs

**Figure 2:**
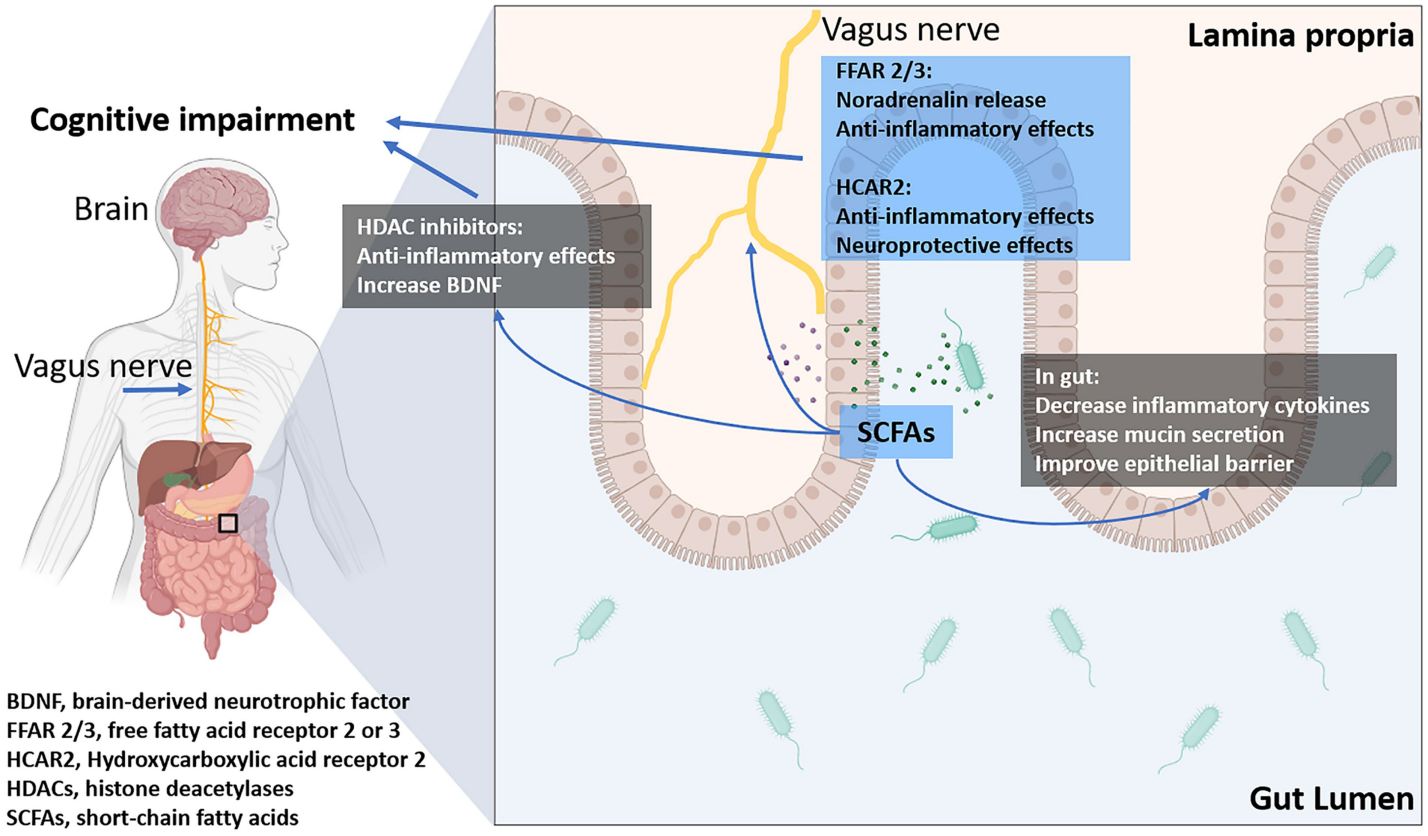
The influence of short-chain fatty acids (SCFAs) on cognitive impairment: SCFAs modulate intestinal inflammation and epithelial barrier function, indirectly affecting disease progression. Additionally, they directly influence the CNS by regulating energy metabolism, neuroinflammation through their receptors, transporters, and histone deacetylases (HDACs).

SCFAs produced by gut microbiota have been shown to influence cognitive function through multiple pathways (Qian et al., 2022). SCFAs, such as acetate, propionate, and butyrate, exert their influence on cognitive function through several mechanisms (Qian et al., 2022). These include modulating the production of neurotransmitters like serotonin, gamma-aminobutyric acid (GABA), and dopamine, which are crucial for mood regulation and cognitive processes (Gasmi et al., 2022). SCFAs stimulate the release of gut hormones, such as glucagon-like peptide-1 (GLP-1), which can cross the blood–brain barrier and affect neuronal signaling (Moțățăianu et al., 2023). Additionally, butyrate has been shown to inhibit histone deacetylase, enhancing gene expression linked to neuroprotection and cognitive health (Jaworska et al., 2017). Butyrate, in particular, enhances synaptic plasticity and memory formation by increasing the expression of brain-derived neurotrophic factor (BDNF) and promoting histone acetylation, which facilitates gene transcription involved in neurogenesis (Li et al., 2022). Butyrate also has anti-inflammatory properties, reducing neuroinflammation and protecting against cognitive decline (Matt et al., 2018; Figure 2).

SCFAs can also modulate the gut-brain axis by influencing the production of gut hormones such as GLP-1 and PYY, which have neuroprotective effects and regulate appetite and energy homeostasis, as previously described (Lai et al., 2024). Furthermore, SCFAs can cross the blood–brain barrier and directly impact brain function by interacting with G-protein-coupled receptors (GPCRs) expressed in the CNS (Frost et al., 2014).

### 5.2 Other microbial metabolites

**Figure 3:**
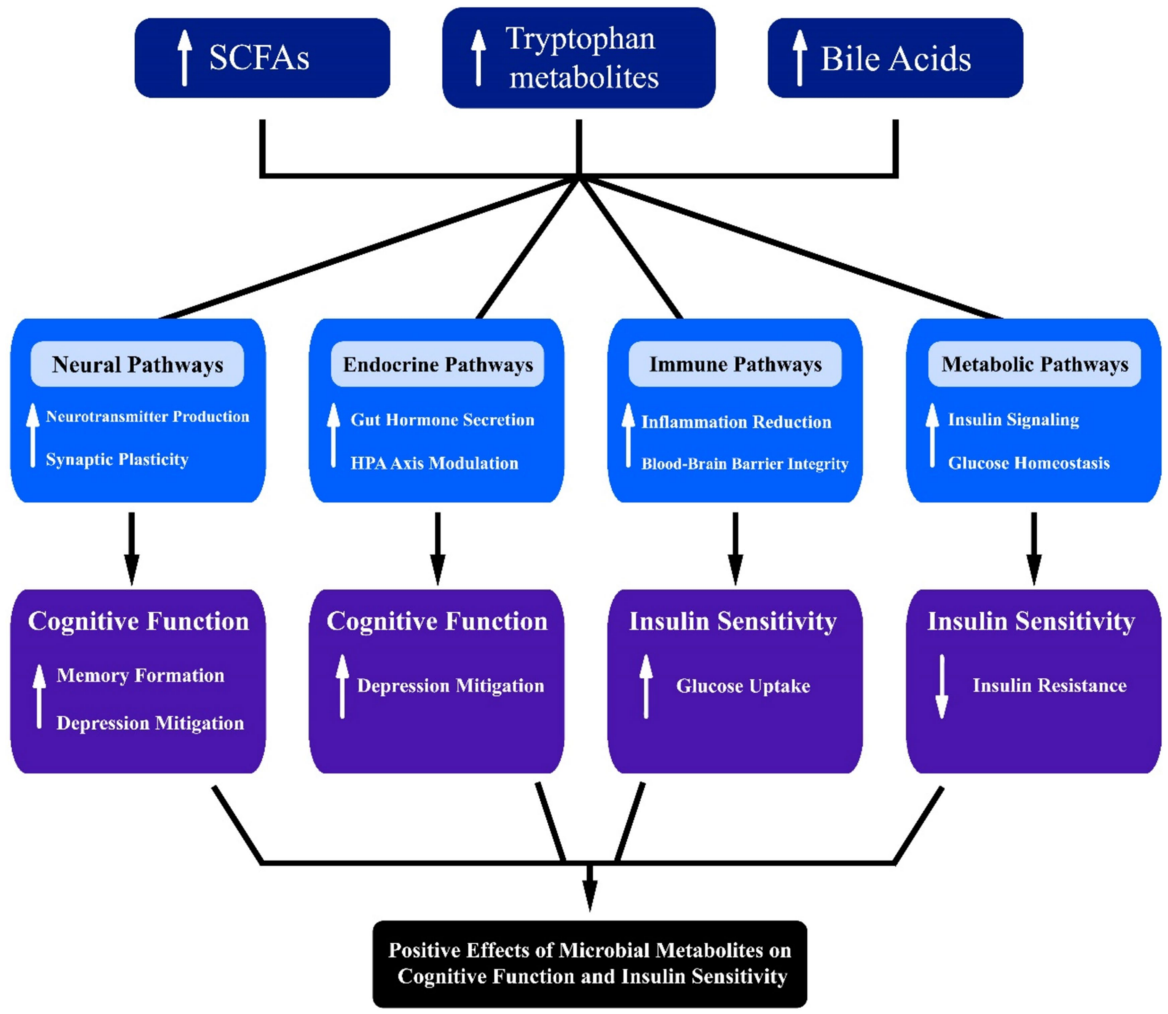
Interconnected pathways illustrating the influence of microbial metabolites, including short-chain fatty acids (SCFAs), tryptophan, and bile acids, on neural, endocrine, immune, and metabolic systems. Arrows denote directional interactions, highlighting potential implications for cognitive function, insulin sensitivity, and metabolic health.

In addition to SCFAs, other microbial metabolites such as tryptophan metabolites, bile acids, and neurotransmitters play significant roles in cognitive function (Figure 3). Tryptophan metabolites, including indole and its derivatives, can modulate the gut-brain axis by interacting with the aryl hydrocarbon receptor (AhR) and influencing immune responses and neuroinflammation (O'Mahony et al., 2015). Alterations in tryptophan metabolism have been linked to depression and cognitive impairment, highlighting the importance of gut microbiota in mental health.

Bile acids, traditionally known for their role in digestion, also function as signaling molecules that can influence brain function. Altered bile acid metabolism has been associated with cognitive decline and neurodegenerative diseases (Baloni et al., 2020). Bile acids can activate TGR5 and FXR receptors in the brain, regulating inflammation and synaptic plasticity (de Aguiar Vallim et al., 2013).

Neurotransmitters produced by gut microbiota, such as gamma-aminobutyric acid (GABA) and serotonin, also significantly impact cognitive function. GABA, an inhibitory neurotransmitter, can help regulate mood and anxiety levels, while serotonin is crucial for mood stabilization and is linked to various cognitive processes. The communication between gut-derived neurotransmitters and the central nervous system may occur through multiple pathways, including direct neural connections and the bloodstream. Recent studies suggest that dysregulation of these neurotransmitter levels can be associated with mood disorders and cognitive impairments, further emphasizing the intricate relationship between gut microbiota and mental health.

## 6 Therapeutic interventions

### 6.1 Probiotics and prebiotics

Probiotics, live microorganisms that confer health benefits to the host, have shown promise in improving insulin sensitivity and cognitive function. Specific strains of probiotics, such as *Lactobacillus* and *Bifidobacterium*, have been shown to reduce inflammation, enhance gut barrier integrity, and modulate metabolic pathways (Wallace and Milev, 2017). Probiotic supplementation can improve glycemic control and reduce markers of insulin resistance in individuals with metabolic disorders (Kootte et al., 2017; Salles et al., 2020).

Prebiotics, nondigestible dietary fibers that promote the growth of beneficial gut bacteria, also have significant potential in modulating the gut-brain-metabolic axis. Prebiotics such as inulin and fructooligosaccharides (FOS) can increase SCFA production, improve gut barrier function, and reduce systemic inflammation (Bindels et al., 2015; Costa et al., 2022). Dietary interventions that incorporate prebiotics can enhance insulin sensitivity and cognitive function by promoting a healthy gut microbiota composition (Deehan and Walter, 2016; Zhang et al., 2019).

### 6.2 Dietary interventions

Diet plays a crucial role in shaping the gut microbiota and influencing the gut-brain axis. High-fiber diets, rich in fruits, vegetables, and whole grains, promote the growth of beneficial gut bacteria and increase SCFA production, which can improve metabolic and cognitive health (Patterson et al., 2016). Conversely, diets high in saturated fats and refined sugars can lead to dysbiosis, increased intestinal permeability, and systemic inflammation, contributing to insulin resistance and cognitive decline (Olson et al., 2018; Baspakova et al., 2024).

Intermittent fasting (IF) has emerged as a dietary intervention with potential benefits for the gut-brain-metabolic axis. IF involves cycles of eating and fasting, which can enhance gut microbiota diversity, reduce inflammation, and improve insulin sensitivity (Mattson et al., 2014; Guzzetta et al., 2022). IF has also been shown to promote neurogenesis and protect against cognitive decline by activating adaptive stress response pathways and increasing BDNF levels (Mattson et al., 2014).

### 6.3 FMT

FMT involves the transfer of gut microbiota from a healthy donor to a recipient with dysbiosis. FMT has shown promise in treating metabolic disorders and improving insulin sensitivity by restoring a healthy gut microbiota composition (Kootte et al., 2017). Emerging evidence suggests that FMT can also improve cognitive function in individuals with neurodegenerative diseases such as Alzheimer’s disease (Baunwall et al., 2020; Tixier et al., 2022).

The therapeutic potential of FMT lies in its ability to reestablish a balanced gut microbiota, reduce systemic inflammation, and enhance gut barrier integrity. However, further research is needed to understand the long-term effects and optimize the protocols for FMT in treating metabolic and cognitive disorders (Baunwall et al., 2020; Tixier et al., 2022).

## 7 Discussion

The interplay between the gut microbiota, insulin resistance, and cognitive function represents a rapidly evolving field with significant implications for health and disease (Kossowska et al., 2024). Future research should focus on longitudinal studies to better understand the causal relationships between gut microbiota composition and changes in insulin sensitivity and cognitive outcomes. Investigating the effects of dietary interventions on gut microbiota, insulin resistance, and cognitive function could provide insights into potential therapeutic strategies. Additionally, exploring the mechanisms by which gut-derived metabolites influence brain function may open new avenues for addressing cognitive decline associated with metabolic disorders.

Key mechanisms involve neural, endocrine, immune, and metabolic pathways (Dinan and Cryan, 2017). The gut microbiota modulates the production of neurotransmitters such as serotonin and dopamine, influences gut-derived hormones like GLP-1, regulates systemic inflammation, and produces metabolites like SCFAs that enhance insulin sensitivity and influence cognitive function (Dicks, 2022). This review highlights the multifaceted mechanisms through which gut microbiota influence metabolic and cognitive processes.

### 7.1 Importance and rationale of this review

This comprehensive review aims to address the aforementioned deficiencies by providing a detailed analysis of the current understanding of the gut-brain-metabolic axis and its implications for insulin resistance and cognitive function. By synthesizing the latest research from various disciplines, including microbiology, neuroscience, and metabolic health, this review was offered a holistic perspective on the complex interplay between the gut microbiota, insulin resistance, and cognitive processes.

The findings of this review will be instrumental in guiding future research directions and informing the development of novel therapeutic interventions. For instance, future studies could explore the potential of targeting specific gut microbial species or their metabolites to improve insulin sensitivity and cognitive function. Additionally, research could focus on designing clinical trials that assess the efficacy of dietary modifications or probiotics in enhancing cognitive health in individuals with insulin resistance. By elucidating these pathways, we aim to pave the way for targeted interventions that leverage the gut-brain axis to mitigate cognitive decline. By elucidating the specific mechanisms underlying the gut-brain-metabolic axis, researchers and clinicians can work toward personalized approaches to prevent and treat metabolic and neurodegenerative disorders, ultimately improving the quality of life for affected individuals.

### 7.2 Novel hypotheses on gut-brain-metabolic axis

#### 7.2.1 Hypothesis 1: multi-strain probiotic synergy

##### 7.2.1.1 Rationale

Current research often focuses on individual probiotic strains. However, considering the complexity of the gut-brain-metabolic axis, a combination of multiple probiotic strains might have synergistic effects (Kwoji et al., 2021).

##### 7.2.1.2 Hypothesis

Administering a multi-strain probiotic cocktail can synergistically enhance SCFA production, improve gut barrier integrity, and modulate neurotransmitter production more effectively than single-strain probiotics, leading to improved insulin sensitivity and cognitive function (Kwoji et al., 2021; Dias et al., 2022).

##### 7.2.1.3 Pathways involved

###### 7.2.1.3.1 Neural

Multi-strain probiotics promote the production of neurotransmitters like serotonin and dopamine via gut microbiota metabolites such as SCFAs. These neurotransmitters are crucial for cognitive processes, including mood regulation and memory function. SCFAs also influence neuroinflammation and the integrity of the blood–brain barrier, impacting cognitive function.

###### 7.2.1.3.2 Endocrine

Increased production of gut hormones (GLP-1, PYY).

###### 7.2.1.3.3 Immune

Reduced inflammation through enhanced SCFA production.

###### 7.2.1.3.4 Metabolic

Improved insulin signaling and glucose homeostasis.

#### 7.2.2 Hypothesis 2: personalized nutrition and microbiota modulation

##### 7.2.2.1 Rationale

Individual variations in gut microbiota composition and metabolic health suggest that personalized dietary interventions could be more effective (Li, 2023).

##### 7.2.2.2 Hypothesis

Personalized nutrition plans based on individual gut microbiota profiles can more precisely modulate gut microbiota composition, enhance SCFA production, and improve cognitive function and metabolic health compared to generalized dietary guidelines (Li, 2023; Kallapura et al., 2024).

##### 7.2.2.3 Pathways involved

###### 7.2.2.3.1 Neural

Personalized diets that enhance precursor availability for neurotransmitters like tryptophan (for serotonin synthesis) can improve gut-brain signaling. Individualized nutrition can also modulate SCFA production, which in turn influences the release of neurotransmitters crucial for cognition.

###### 7.2.2.3.2 Endocrine

Diet-induced modulation of gut hormone levels.

###### 7.2.2.3.3 Immune

Personalized nutrition reducing individual-specific inflammatory responses.

###### 7.2.2.3.4 Metabolic

Enhanced insulin sensitivity through targeted dietary components.

#### 7.2.3 Hypothesis 3: microbiota-derived exosome therapy

##### 7.2.3.1 Rationale

Exosomes are extracellular vesicles that play a crucial role in intercellular communication. Gut microbiota-derived exosomes could carry bioactive compounds that influence distant organs, including the brain (Manzaneque-López et al., 2023; Díez-Sainz et al., 2021; Zhang B. et al., 2022; Zhao et al., 2021).

##### 7.2.3.2 Hypothesis

Microbiota-derived exosome therapy can be developed to deliver specific microbial metabolites directly to the brain, enhancing cognitive function and insulin sensitivity by modulating the gut-brain-metabolic axis (Manzaneque-López et al., 2023; Díez-Sainz et al., 2021; Zhang B. et al., 2022; Zhao et al., 2021). However, several technical and safety challenges must be addressed to realize this therapeutic potential.

To overcome technical challenges, researchers should focus on optimizing exosome isolation and characterization methods to ensure purity and functionality. Advanced techniques such as ultrafiltration and size-exclusion chromatography can improve the yield and quality of exosomes. Moreover, developing robust delivery systems that protect exosomes from degradation and facilitate their transport across the blood–brain barrier is crucial.

Regarding safety challenges, comprehensive toxicological studies are essential to assess the biocompatibility and potential immunogenicity of microbiota-derived exosomes. Conducting preclinical trials in relevant animal models will help evaluate the therapeutic efficacy and safety profile before progressing to clinical trials. By addressing these challenges, microbiota-derived exosome therapy may become a viable strategy for enhancing cognitive function and metabolic health.

##### 7.2.3.3 Pathways involved

###### 7.2.3.3.1 Neural

Exosomes from microbiota can carry bioactive compounds, including neurotransmitters, peptides, and other neuroprotective molecules, across the gut-brain axis. This could influence brain function by modulating synaptic plasticity, reducing oxidative stress, and supporting neurogenesis, ultimately improving cognitive function and insulin sensitivity.

###### 7.2.3.3.2 Endocrine

Regulation of HPA axis via exosome-contained signaling molecules.

###### 7.2.3.3.3 Immune

Exosomes reducing systemic inflammation by inhibiting pro-inflammatory cytokine production, thereby improving blood–brain barrier integrity and preventing neuroinflammation.

###### 7.2.3.3.4 Metabolic

Enhanced insulin signaling and metabolic regulation through targeted exosome delivery.

#### 7.2.4 Hypothesis 4: microbiota-gut-brain peptide modulation

##### 7.2.4.1 Rationale

Peptides produced by gut microbiota can influence gut-brain communication and metabolic processes (Lai et al., 2024; Sun et al., 2020).

##### 7.2.4.2 Hypothesis

Enhancing the production of specific gut-derived peptides through dietary or probiotic interventions can improve gut-brain communication, reduce neuroinflammation, and enhance insulin sensitivity and cognitive function (Lai et al., 2024; Sun et al., 2020).

##### 7.2.4.3 Pathways involved

###### 7.2.4.3.1 Neural

Gut-derived peptides can enhance the release of neurotransmitters and support synaptic plasticity, crucial for learning and memory. For example, the gut peptide ghrelin has been shown to influence hippocampal function and cognitive performance, while other microbiota-derived peptides may reduce neuroinflammation.

###### 7.2.4.3.2 Endocrine

Modulation of gut-brain peptides impacting hormone release.

###### 7.2.4.3.3 Immune

Anti-inflammatory peptides reducing systemic and neuroinflammation.

###### 7.2.4.3.4 Metabolic

Peptides enhancing insulin signaling pathways.

### 7.3 Therapeutic potential

Therapeutic interventions such as probiotics, prebiotics, dietary modifications, and FMT offer promising avenues for modulating the gut-brain-metabolic axis. Probiotics have been shown to increase the production of SCFAs, such as butyrate, which improve gut barrier integrity, reduce neuroinflammation, and enhance insulin sensitivity. SCFAs also cross the blood–brain barrier, impacting neurotransmitter synthesis and modulating cognitive function. Prebiotics selectively stimulate the growth of beneficial bacteria, thereby influencing metabolic pathways related to glucose homeostasis and neuroprotection. FMT, by restoring a balanced microbiota, can reduce systemic inflammation, restore insulin signaling, and improve cognitive performance by promoting the production of neuroactive metabolites. The efficacy of these interventions in improving insulin sensitivity and cognitive function underscores the potential for personalized approaches to prevent and treat metabolic and neurodegenerative diseases (Kootte et al., 2017; Matt et al., 2018). However, further research is needed to optimize these interventions, understand their long-term effects, and identify the most effective strategies for different populations.

### 7.4 Limitations of current therapeutic interventions

While therapeutic interventions targeting the gut-brain-metabolic axis, such as probiotics, prebiotics, dietary modifications, and FMT (Contarino et al., 2023), show promise in regulating insulin resistance and cognitive function, several limitations and unresolved issues warrant attention:

#### 7.4.1 Variability in individual response

One of the significant challenges is the variability in individual responses to these interventions (Almeida et al., 2022). Factors such as baseline gut microbiota composition, genetic predispositions, dietary habits, and lifestyle can influence how individuals respond to therapeutic strategies (Strasser et al., 2021). This variability complicates the predictability of outcomes, making it difficult to generalize findings across populations.

#### 7.4.2 Lack of standardization

Many probiotic products on the market lack standardization concerning strain composition, dosage, and delivery methods (Zawistowska-Rojek et al., 2022). This inconsistency can lead to varying efficacy and safety profiles across different studies, limiting the ability to draw definitive conclusions about their effectiveness in modulating the gut-brain-metabolic axis (Ullah et al., 2021).

#### 7.4.3 Short-term study duration

Most clinical trials assessing the efficacy of therapeutic interventions are relatively short-term, often ranging from a few weeks to a few months (Biazzo and Deidda, 2022). This limited duration raises concerns about the sustainability of benefits observed and the potential emergence of adverse effects over longer periods of use (Biazzo and Deidda, 2022).

#### 7.4.4 Mechanistic insights

There is still a lack of clear mechanistic understanding of how specific interventions exert their effects on the gut-brain-metabolic axis (O'Riordan et al., 2022). Without a robust understanding of the underlying biological pathways, it is challenging to optimize these therapies for maximum efficacy and safety (Chakrabarti et al., 2022).

#### 7.4.5 Safety concerns

The long-term safety of interventions, particularly FMT, remains an unresolved issue (Dang et al., 2020). Concerns about the transfer of pathogenic microorganisms, alterations in gut microbiota composition, and unintended metabolic effects necessitate further investigation to ensure patient safety (Matzaras et al., 2022).

#### 7.4.6 Regulatory challenges

Regulatory frameworks for probiotics and other microbiota-targeted therapies can be inconsistent, leading to disparities in product quality and safety (Merenstein et al., 2023). This inconsistency can hinder the advancement of effective therapies in clinical settings.

By addressing these limitations, future research can focus on developing more effective, personalized, and safer therapeutic interventions targeting the gut-brain-metabolic axis. Further studies should aim to elucidate the specific microbial species and metabolites involved, evaluate long-term effects, and establish standardized protocols to enhance the clinical applicability of these interventions.

### 7.5 Challenges and controversies in researching the gut-brain-metabolic axis

Despite the promising findings regarding the role of gut microbiota in regulating insulin resistance and cognitive function, several challenges and controversies remain in this field:

#### 7.5.1 Variability in microbiota composition

Individual variations in gut microbiota composition can lead to inconsistent results across studies. Factors such as diet, genetics, environment, and lifestyle can significantly influence microbial diversity, making it difficult to establish universal conclusions.

#### 7.5.2 Causality vs. correlation

Much of the current research highlights correlations between gut microbiota and metabolic/cognitive outcomes. However, establishing causality remains a challenge, as it is unclear whether changes in gut microbiota directly cause metabolic and cognitive changes or if they are simply a consequence of these conditions.

#### 7.5.3 Methodological differences

The diverse methodologies employed in studies, including variations in sampling techniques, analytical methods, and experimental designs, can lead to disparate findings. This lack of standardization complicates the interpretation and comparison of results.

#### 7.5.4 Mechanistic understanding

While there are hypotheses about the mechanisms through which gut microbiota affect insulin resistance and cognitive function, more research is needed to elucidate these pathways fully. Understanding the specific microbial species and metabolites involved in these processes is crucial for advancing therapeutic approaches.

#### 7.5.5 Safety and efficacy of interventions

As therapeutic strategies targeting gut microbiota, such as probiotics and FMT, gain attention, concerns about their long-term safety and efficacy need to be addressed. More rigorous clinical trials are necessary to evaluate the potential risks and benefits of these interventions.

### 7.6 Future directions

Future research should focus on elucidating the specific microbial species and metabolites involved in modulating insulin resistance and cognitive function. To achieve this goal, researchers could employ high-throughput sequencing techniques to identify and characterize gut microbiota in diverse populations, particularly those with varying degrees of insulin resistance and cognitive impairment. Additionally, metabolomic analyses can be utilized to profile metabolites produced by these microbial communities. Integrating these approaches with clinical studies will allow for the exploration of causal relationships and the identification of potential biomarkers for early intervention. Understanding these specific interactions will be crucial for developing targeted therapies aimed at improving metabolic and cognitive health.

Advances in metagenomics, metabolomics, and bioinformatics will enable a deeper understanding of the complex interactions within the gut microbiota and their impact on host health (Zhang Q. et al., 2022; Takeuchi et al., 2023). Additionally, exploring the role of the gut-brain-metabolic axis in various disease states, including neurodegenerative diseases, psychiatric disorders, and metabolic syndromes, will provide valuable insights into the underlying mechanisms and potential therapeutic targets (Ridaura et al., 2013; Kootte et al., 2017).

Moreover, the long-term effects and safety of therapeutic interventions targeting the gut-brain-metabolic axis, such as probiotics, prebiotics, and FMT, are not well-established. Longitudinal studies are necessary to evaluate the sustainability and potential adverse effects of these interventions (Fekete et al., 2024; Makris et al., 2021).

### 7.7 Future prospective

The gut-brain-metabolic axis represents a promising area of research with significant implications for understanding and treating metabolic and cognitive disorders. Emerging evidence suggests that targeting the gut microbiota through dietary interventions, probiotics, prebiotics, and FMT can improve insulin sensitivity and cognitive function, offering new avenues for therapeutic strategies.

Future research should focus on identifying the specific microbial species and metabolites that play critical roles in modulating the gut-brain axis and their impact on metabolic and cognitive health. Advances in sequencing technologies and bioinformatics will enable a more comprehensive understanding of the gut microbiota and its interactions with the host.

Additionally, personalized approaches that consider individual variations in gut microbiota composition, genetics, and lifestyle factors will be crucial for optimizing therapeutic interventions. Understanding the long-term effects and safety of these interventions, particularly FMT, will be essential for their clinical application (Kootte et al., 2017; Mattson et al., 2014).

Exploring the role of the gut-brain-metabolic axis in various disease states, including neurodegenerative diseases, psychiatric disorders, and metabolic syndromes, will provide valuable insights into the underlying mechanisms and potential therapeutic targets. Ultimately, a better understanding of the gut-brain-metabolic axis will pave the way for novel strategies to promote metabolic and cognitive health, improving the quality of life for individuals affected by these interconnected disorders (Arnold et al., 2018).

## 8 Conclusion

The gut-brain-metabolic axis is a promising area of research with significant implications for understanding and treating metabolic and cognitive disorders. This review uniquely synthesizes the latest findings on the interplay between gut microbiota, insulin resistance, and cognitive function, providing a comprehensive overview of the mechanisms involved. Unlike previous studies, this review not only highlights key microbial species and metabolites but also identifies gaps in current knowledge and proposes targeted research directions. By integrating insights from multiple disciplines, we aim to facilitate the development of novel therapeutic strategies that leverage the gut-brain axis to improve metabolic and cognitive health. Our findings underscore the necessity for future studies to explore the clinical applications of these insights, ultimately contributing to the advancement of personalized medicine in metabolic and cognitive disorders. Targeting the gut microbiota through dietary, probiotic, and fecal transplant interventions has the potential to improve insulin sensitivity and cognitive function. Future studies should focus on identifying the key microbial species and metabolites involved, evaluating long-term intervention effects, and exploring the axis’ role in diverse disease states. Personalized approaches considering individual variations will be crucial. Ultimately, this research can lead to novel strategies to promote metabolic and cognitive health.
